# A modeling study of the impact of treatment policies on the evolution of resistance in sea lice on salmon farms

**DOI:** 10.1371/journal.pone.0294708

**Published:** 2023-11-29

**Authors:** Enrico Trombetta, Sara Jakubiak, Sara Kutkova, Debby Lipschutz, Anthony O’Hare, Jessica A. Enright

**Affiliations:** 1 School of Computing Science, University of Glasgow, Glasgow, United Kingdom; 2 Usher Institute, University of Edinburgh, Edinburgh, United Kingdom; 3 Computing Science and Mathematics, University of Stirling, Stirling, United Kingdom; Universitat Politècnica de València, SPAIN

## Abstract

Salmonid aquaculture is an important source of nutritious food with more than 2 million tonnes of fish produced each year (Food and Agriculture Organisation of the United Nations, 2019). In most salmon producing countries, sea lice represent a major barrier to the sustainability of salmonid aquaculture. This issue is exacerbated by widespread resistance to chemical treatments on both sides of the Atlantic. Regulation for sea lice management mostly involves reporting lice counts and treatment thresholds, which depending on interpretation may encourage preemptive treatments. We have developed a stochastic simulation model of sea lice infestation including the lice life-cycle, genetic resistance to treatment, a wildlife reservoir, salmon growth and stocking practices in the context of infestation, and coordination of treatment between farms. Farms report infestation levels to a central organisation, and may then cooperate or not when coordinated treatment is triggered. Treatment practice then impacts the level of resistance in the surrounding sea lice population. Our simulation finds that treatment drives selection for resistance and coordination between managers is key. We also find that position in the hydrologically-derived network of farms can impact individual farm infestation levels and the topology of this network can impact overall infestation and resistance. We show how coordination and triggering of treatment alongside varying hydrological topology of farm connections affects the evolution of lice resistance, and thus optimise salmon quality within socio-economic and environmental constraints. Network topology drives infestation levels in cages, treatments, and hence treatment-driven resistance. Thus farmer behaviour may be highly dependent on hydrologically position and local level of infestation.

## 1 Introduction

Atlantic salmon (*Salmo salar*) is a commonly farmed fish and contributed £614M to UK exports in 2021 and $13.1bn globally [1]. Farmed salmon are kept in large sea cages and are susceptible to infestation from a number of natural parasites which proliferate in part due to high stocking densities and their offspring are subsequently carried on sea currents to infest other cages. The most economically important parasite in Scotland is the sea louse (*Lepeophtheirus salmonis*) [2–4].

Infestation can cause stress, secondary infections and physical damage to the fish [2] due to the lice feeding on skin, mucus, and blood [5, 6] and control methods can incur considerable expense to farmers [7]. Infestation causes health and welfare issues for the salmon, leading to lower weight gain and thus lower prices for the farmer and in the most serious cases, mortality in the fish. Effective control of sea lice is therefore important for profitability and animal welfare on salmon farms, but also avoid spillover to wild salmonids [3, 4]. Infestation of wild salmon may be mitigated by predation [8] or migration [9, 10], but these are not typically possible for farmed salmon.

There are a number of treatments that are used to treat salmon for sea lice once an infestation is detected. Veterinary drugs, which we will refer to as “chemical treatments”, have been employed for the last 50 years [11, 12]. Currently available delousing agents licensed in Scotland belong to five drug classes, namely organophosphates (e.g. azamethiphos, the active ingredient in Salmosan, are water soluble and broken down relatively quickly in the environment, causes paralysis and death in sea-lice), pyrethoids (e.g. Cypermethrin and deltamethrin, stimulate the sodium channels in neuronal cells, inducing paralysis leading to death in sea-lice without affecting salmon), avermectins (are the major drugs used as in-feed treatments), hydrogen peroxide (a bath treatment that knocks sea lice off fish leaving them free to attach again, can be toxic to fish) and benzoylureas (inhibits synthesis of chitin during molting of immature stages of louse, but are thought to have adverse effects on non-target species such as crustaceans and amphipods)[13]. However, as *L. Salmonis* are evolving resistance successfully to such treatments, purely chemical-based treatment schedules are proving unsustainable. Genetic resistance to these treatments is not mutually exclusive, meaning the development of pan-resistant lice is a real possibility and threat to the market [13, 14]. Furthermore, these treatments pose environmental challenges such as mollusc communities close to the fish farm location [15, 16] and references therein.

A second approach is presented by mechanical treatments that rely on physical, automated delousing operations [17]. In principle, physical delousers apply some force that physically detaches the lice from the fish skin, either by pressured water jets (hydrolicers) or brief, hot baths (thermolicers). Both thermolicers and hydrolicers have been used in Scotland since 2016, and in Norway they have become the preferred method of delousing salmon [12].

Such delousing treatments may cause increased stress levels in comparison to chemical treatments due to the insurgence of bruises, haemorrhage, pain and consequently fish death. The efficacy of thermolicer treatment fluctuates depending on previous lice exposure to high temperatures.

A third approach is offered by biological cleaner fish, such as lumpsucker (*Cyclopterus lumpus*) and ballan wrasse (*Labridae*) [18, 19]. These constitute the most environmentally friendly approach to deinfestation, although their efficacy is reportedly not on par with conventional methods.

Farms may also lie fallow, where sites are left empty for a period to decrease lice prevalence.

All treatments have costs in economic terms, fish damage (intoxication, increased stress levels etc.) and/or ecological impact and there is now extensive proof that genetic resistance to treatment develops [20–22].

There are a number of key factors in the biology of the sea louse that make its evolution particularly sensitive to our control strategies. sea lice are short lived but have a high fecundity [18, 23, 24] and their larvae are dispersed over a wide area by ocean currents. They display a wide range of genetic traits including tolerance to salinity and heat [25]. The number of available hosts in farm cages far exceed the number of wild hosts [3] and when multiple farms in a region apply the same treatment, a large selection pressure is exerted leading to population level resistance which has been seen in the level of resistance such as the resistance to delousing chemicals observed across the north Atlantic [26, 27].

A number of models have been developed to model the lifecycle of sea lice, through their mobile development stages (copepodid, chalimus), pre-adult stages and adult stages [17, 28–30]. An ordinary differential equation model determining the growth of lice through different stages (compartments) was developed by Stein [28] which included temperature dependent development times.

The same sea lice life stages were used in a stochastic model of the Norwegian salmon industry by [29] that allows for stage-age evolution and treatment mortality (more treatments were included by [17]). In this model, each farm consists of several sea cages stocked with salmon and subject to free floating lice (egg and copepod stages) and infestation by adult lice. Treatment induced mortality in salmon was included by [30]. However, neither of these models incorporate treatment resistance.

A similar sea lice life-cycle model was developed for the Scottish salmon industry to compare different treatments [31]. In Scotland, salmon farms are managed through local collaborative agreements. These farm management areas are required to control notifiable diseases and pest outbreaks through coordinated treatments but such agreements can lack legal force [32, 33].

Some recent models have explored the effect of a fitness cost associated with resistance, showing that it can slow down the evolution to resistance, particularly when wild salmon populations can act as refugia [34] and that resistance is most strongly selected under moderate levels of treatment on farms [35].

In this paper we generalise the previous models described above [17, 28–32, 34, 35], allowing for modelling of the Scottish salmon industry and by adding a genotype distribution for the lice population to model treatment resistance. We included a hierarchical arrangement of sea cages in farms which in turn are part of a management collective allowing coordination in the use of chemicals (the most common chemical treatment in salmon salmon aquaculture is Emamectin Benzoate, EMB). Our model incorporates the same sea lice lifecycle as previous models, infestation and treatment including treatment mortality of lice and fish.

Our model includes treatment decisions that are made at the individual farm level, at a broader industry level, or may be dictated by regulation. We encapsulate our model in a graphical game determining the evolution of resistance as a consequence of the location of the farm and the degree of collaboration in treatments.

### 1.1 Contribution

We have developed an open-source stochastic model of sea lice development, infestation, and treatment with evolving resistance across a number of salmon farming sites linked by hydrology.

We simulate populations of salmon and lice via distributions of numbers and lice genetics rather than as individuals, thus allowing us to simulate a whole sea loch containing several farms with relatively low memory requirements. We incorporate our lice and treatment model into a game theoretic model where each agent (farmer) seeks to maximise their own profit and may choose to collaborate or not with neighbours on the choice and timing of treatments.

Importantly, our model includes explicit encoding of evolution of resistance to treatments. In our model, repeated use of chemicals to control sea lice creates a selection pressure that drives evolution of tolerance and eventually resistance to chemical treatments. We are thus able to investigate the impact of different treatment coordination strategies on the evolution of resistance.

## 2 Materials and methods

A salmon *cooperative* manages a number of farms, each farm containing several cages which are operational for 18 months to 2 years and subject to environmental pressure of sea lice (free swimming sea lice in the loch). We simulate the development of lice and salmon, infestation of salmon, mortality of sea lice and salmon (including treatment induced mortality), on a daily time step over several years using a model similar to [17, 29, 30] but with genotype information for the sea lice population related to resistance to treatment, and explicitly include this in treatment impact calculations and reproduction events. We model several treatments and treatment-cooperation policies between farms.

This section will give an outline of our model and its data. Further details are given in the S1 File.

### 2.1 Data

#### 2.1.1 Location data and farm connection network

We simulate two sea lochs in western Scotland, Loch Fyne (hosting 9 farms) and Loch Linnhe (hosting 10 farms). Site data for location, number of cages, their capacity, and available facilities are extracted from Marine Scotland’s Data Portal [36].

Sea temperature at each farm was interpolated from the mean sea temperature from two sea monitoring stations at Ardrishaig and Tarbert, www.seatemperature.org, using the farms geographical location.

We used wind speeds and average dispersion rates between farms from the Scottish Shelf Model to create a matrix of the likelihood of a louse egg travelling between farms [37]—this produces the network of farm linkages.

#### 2.1.2 Biological data

We extracted sea lice counts, fish mortality reports, treatment application and/or other lice control decisions from available Scottish reports available from Marine Scotland, the Scottish Salmon Company. Due to a change in format and frequency in 2015 and 2021, we consider monthly updated reports over the period between January 2016 and December 2018 [36, 38].

We used Bayesian Optimization (BayesOpt) [39] bundled with Ray Tune to determine several parameters for our model from monthly published adult female lice counts per fish and fish mortality counts (see S1 File). An advantage of BayesOpt compared to Bayesian MCMC is that it does not require an explicit definition to minimise, while providing similar abilities to explore the state space in potentially high dimensions and climb local optima.

The constants in our expressions for transitions between lice life stages were determined by fitting to reports from Salmon Scotland and Marine Scotland using [39].

We fit a simple logistic curve to FAO data [40] to model the growth of salmon, a simple quadratic curve to model fish mortality using the data in [17], and a non-linear function to model lice mortality for thermolicer and hydrolicer treatments from [41].

### 2.2 Salmon and sea lice development

This subsection contains only an outline of our implemented model of salmon and lice development, which in general follow those in [29]. We include fuller details in the attached S1 File.

#### 2.2.1 Lice stages

The life cycle of the sea louse undergoes as many as 14 transformational stages [31]. We use a simplified life cycle, identifying 5 major stages similar to the approach taken by [28, 29, 42, 43]:

1. *Nauplius (R)*: The sea lice nauplii hatch from eggs and drift in the water with some very limited ability to move. This stage is also called planktonic.
2. *Copepodid (CO)*: Nauplii develop further into copepodids. They also drift in water and have limited movement but can perform initial attachment to the fish (infestation). A sea louse needs to be attached to a fish to develop further into the chalimus.
3. *Chalimus & Pre-adult (CH & PA)*: Chalimus performs further attachment to a fish. They develop into pre-adult sea lice and are now mobile and can move on the host and swim in the water column. They cannot yet reproduce. The literature commonly separates the chalimus stage into I and II but for our modelling, a single stage with longer evolution times is sufficient as the observable behaviour between the two sub-stages is indistinguishable. Similar considerations apply to Pre-adult I and II stages.
4. *Adult (A)*: Adult sea lice, further split into adult male (L5m) and adult female (L5f), can reproduce if they are attached to the same salmon.

The number of lice in each stage depends on a number of factors, e.g. water temperature (warmer sea temperature promotes development), salinity [44], external pressure as a function of time, egg hatching, development through each life stage, and natural and treatment induced mortality. At each step in our simulation we calculate the number of sea lice in each stage by evolving lice through the life stages and determining the number that die naturally (see Table 2 in S1 File)) or through treatment-induced mortality. Natural lice mortality is assumed to be a simple constant $\mu_{b}^{SAL}$, calculated from the Scottish fish farm production survey 2016 [38].

For the first nauplius stage, lice can easily float around cages but for simplicity this is not modelled as we assume all the cages will contain the same number of fish thus re-circulation effects in nearby cages would cancel out. We model the external pressure as an infinite, dynamic generator of new lice.

The evolution of lice from the copepod to chalimus stage is only possible if the louse has managed to attach to a salmon, those that do not attach die.

#### 2.2.2 Lice mating and reproduction

Mating occurs between adult lice on the same host fish and the probability of successful mating and egg production is calculated using the methods in [29, 42]. We model the time to egg hatching using a regression model from [28]. Details of the mating and hatching distributions and parameters can be found in the S1 File.

The majority of lice hatching from eggs are typically lost, some are transported to neighbouring farms via sea currents and few are reintegrated into the reservoir (the sea loch where they can potentially be used as sources of lice in the future). Knowing the locations of each farm we calculate the Euclidean distance between pairs of farms, *d*_*ij*_, and the probability that an egg leaving farm, *i*, reaches farm *j*, *r*_*ij*_ from [37], and the time taken to reach the destination is Poisson-distributed.

We maintain a genotype distribution in the lice population for the genes that confer resistance to the different treatment types available (making the simplifying assumption that a single genotype confers resistance to treatment). Our genotype is modelled as recessive (aa), homozygous dominant (AA), or heterozygous dominant (Aa). During mating these alleles are combined according to a simple Mendelian approach, taking one gene from each adult for the offspring.

#### 2.2.3 Fish growth and mortality

We fit a simple logistic curve to Food and Agriculture Organisation of the United Nations (FAO) data [40] to model the growth of salmon in cages (in kg).

Few models capture fish mortality, either assuming no background mortality, lice infestation being an environmental threat, or disregarding treatment effect. Lice on farmed fish do not typically cause host death directly parasite load is extremely high over a long period of time, but can provide a route for other potentially-lethal pathogens to infect the fish. Because of regulation on Scottish aquaculture producers on allowed lice numbers on fish [45] treatment and culling are more likely causes of death.

In our model we include several modes of fish mortality. Background (natural) mortality is considered irrespective of the genotype of attached lice, meaning each allele is equally likely to be removed. Mortality from lice infestation—which as above is very rare—is given by [30]. Thus the number of salmon in a cage slowly decreases over the simulation period. We do not top-up cages with new salmon.

Fish mortality due to attached sea lice depends uniquely on pathogenic lice load, which comprises PA and A stage. We use a simple sigmoid-based function for the mortality due to the number of attached lice similar to Vollset [30].

#### 2.2.4 Treatment

Three different classes of treatments are modelled within SLIM; chemical treatment (here we model lice mortality due to EMB similar to [29]), mechanical treatments (we fit a non-linear function for lice mortality due to thermolicer treatment from [41]) and biological treatments (cleaner fish, where lice mortality is modelled as a decaying exponential similar to [29]). Details of how the different treatments are modelled and how the genotypes of the lice affect the efficacy of treatment are given in the S1 File.

We also allow farms to lie fallow, i.e. contain no fish; this is enforced if the mean number of attached lice is greater than a prescribed level.

Treatments are responsible for much of the lice and fish mortality, thus requiring caution when using them.

### 2.3 Treatment policies

The main goal of this work is to detect how changes in the timing or choice of treatment may drive better results. We embed our model in a game theoretic framework that partly mimics existing regulations for Scottish aquaculture.

Each farm calculates the number of lice in each cage in each stage, attached lice, treatments being applied, and fish population daily. The state of each farm is available to the organisation who determines whether or not a treatment should be applied globally.

When the organisation declares that a treatment should be applied globally, farms will perform a single action from the following (in order of increasing severity) depending on its state and any directives from an organisation: no action, add cleaner fish to cages, chemical treatment (EMB), thermolicer, or fallowing. This choice of action is dependent on the particular policy being applied.

We restrict the number and types of treatments that can be applied to mimic current regulations: with the exception of cleaner fish, which can be applied without restriction, no more than 10 treatments can be executed in a calendar year, to protect fish health and the environment. If EMB is being applied on a farm or is currently active, it cannot be re-applied. If the mean number of attached lice is greater than 6 per adult fish for 4 consecutive weeks, the farm is culled and forced to lie fallow.

We do not allow for collaborative strategies such as tit-for-tat etc. but farms are allowed to defect from organisational dictated actions in some experimental policies. Each farms payoff function takes into account the daily potential profit one could gain from salmon, discounted by damage done by pathogenic lice and treatment costs.

We consider two extreme policies over the whole system, and two variants of more realistic policies. Our extreme whole-system policies are essentially straw men for comparison to our more moderate and realistic policies, and are a zero-treatment policy where no treatment is applied by any farm, and cooperative regular treatment where treatment by EMB is applied at regular scheduled intervals across the whole system regardless of lice burden.

Our two policies more realistic policies are:

- **Bernoullian** a policy with some fixed probability of defection and random treatment choice (we refer to this as a ‘Bernoullian’ policy as this describes the simple uniform probability approach for choosing whether or not to defect). Under our Bernoullian policy a farm that does not itself exceed the lice treatment threshold, when receiving a notification from the organisation that it ought to treat, will choose not to treat with some fixed defection probability. If it does treat, it chooses a random treatment option that respects the rules on treatment repeats. Any farm that itself exceeds the lice threshold for treatment will not defect, and will always treat when told to. The main parameter of our Bernoullian treatment policy is the probability of defection, *p*, that when a treatment suggestion is triggered by the regional organisation, an individual farm ignores the suggestion to treat. Thus when *p* = 1.0 farms will only treat when they themselves are above the lice aggregation threshold that triggers treatment, but not when this trigger is caused by another farm on the loch, and when *p* = 0.0 a farm will treat every time the treatment trigger is suggested. In our experiments every farm has the same *p*: we leave an examination of heterogeneous probability settings as important future work.
- **Mosaic** In the mosaic treatment regime farms trigger a treatment in response to an organisation-wide suggestion (when the lice count in one of the farms exceeds a specified threshold), and that all farms obey this suggestion, there is no defection. However, in contrast to the Bernoullian setting, which does not prescribe treatment types, when following the mosaic policy, farms cycle through the available treatments in a pre-specified order.

### 2.4 Farm instantaneous payoff estimation

A payoff function *r*_*t*_ = *r*(*s*_*t*_) takes into account the daily potential profit one could gain from salmon (projected weight multiplied by the price per kilo) with a discount for attached lice and subtracting treatment costs. This payoff is not used by farms to choose actions, but instead as a means of our recording the benefits of any particular policy.

$$\begin{array}{cc} r_{tfc} & =k_{r}[W_{tfc}N^{SAL}-k_{ar}\left(N^{path}/N^{SAL}\right)]-\sum_{T}\left(\chi_{tfc}^{T}k_{T}\right)\\ r_{t} & =\sum_{f}\sum_{c}r_{tfc} \end{array}$$

where *k*_*r*_ is a conversion rate between fish weight and market price (unit: £/Kg), *k*_*ar*_ is a discounting factor depending on the lice aggregation rate, and *k*_*T*_ is the cost of application of the treatment *T*. *N*^*path*^ being the number of pathogenic lice (assumed to be all mobile stages but CH), $\chi_{tfc}^{T}$ the treatments applied to cage *c* on farm *f* at time *t*.

### 2.5 Simulation framework

We run repeated trials of our simulation to assess the importance of farm connectivity and treatment policies on lice population, fish-related farm payoff, and levels of lice resistance.

The simulator was written in Python with SciPy, NumPy, and Scikit Learn numerical processing libraries. The package supports multi-processing mode for single runs or parallel runs.

Our experiments benchmarking each policy were run on a server node running Ubuntu 20.04 with dual Intel Xeon E5–2697A v4 CPUs and 512GB RAM.

## 3 Results and discussion

In our experiments the following policies have been evaluated:

- Zero treatment policy, as a baseline to compare the efficacy of different policies.
- Fully-cooperative regular treatment policy across all farms where treatments are applied at regular specified time intervals.
- Mosaic treatment policy, with a different globally-mandated treatment being applied in a regular predetermined order with full farmer cooperation.
- Bernoullian treatment policy, where farmers may *defect* from the globally-mandated treatment with a specified probability *p*.

We include experiments on three types of farm network topology, one type derived from data and the other two synthetic for proof-of-concept experiments showing that topology can play a role:

- Loch Fyne, Loch Linnhe: Sea lochs on the west coast of Scotland. We use the eastings and northings locations of farms on these lochs as the locations of the nodes/farms within our simulation with edge weights derived from hydrological linkages.
- Clique: Every farm in the simulation is pairwise connected. Since each farm in a loch is connected to all others in the loch sea-lice can migrate to every farm in the loch.
- Path: Farms are arranged in a linear path with each farm connected only to its neighbours in the path so that sea-lice can only migrate to one neighbouring farm due to simulated hydrological conditions in the loch.

The results of the synthetic networks are presented in section 3.4.

### 3.1 Extremes of treatment

**Fig 1:**
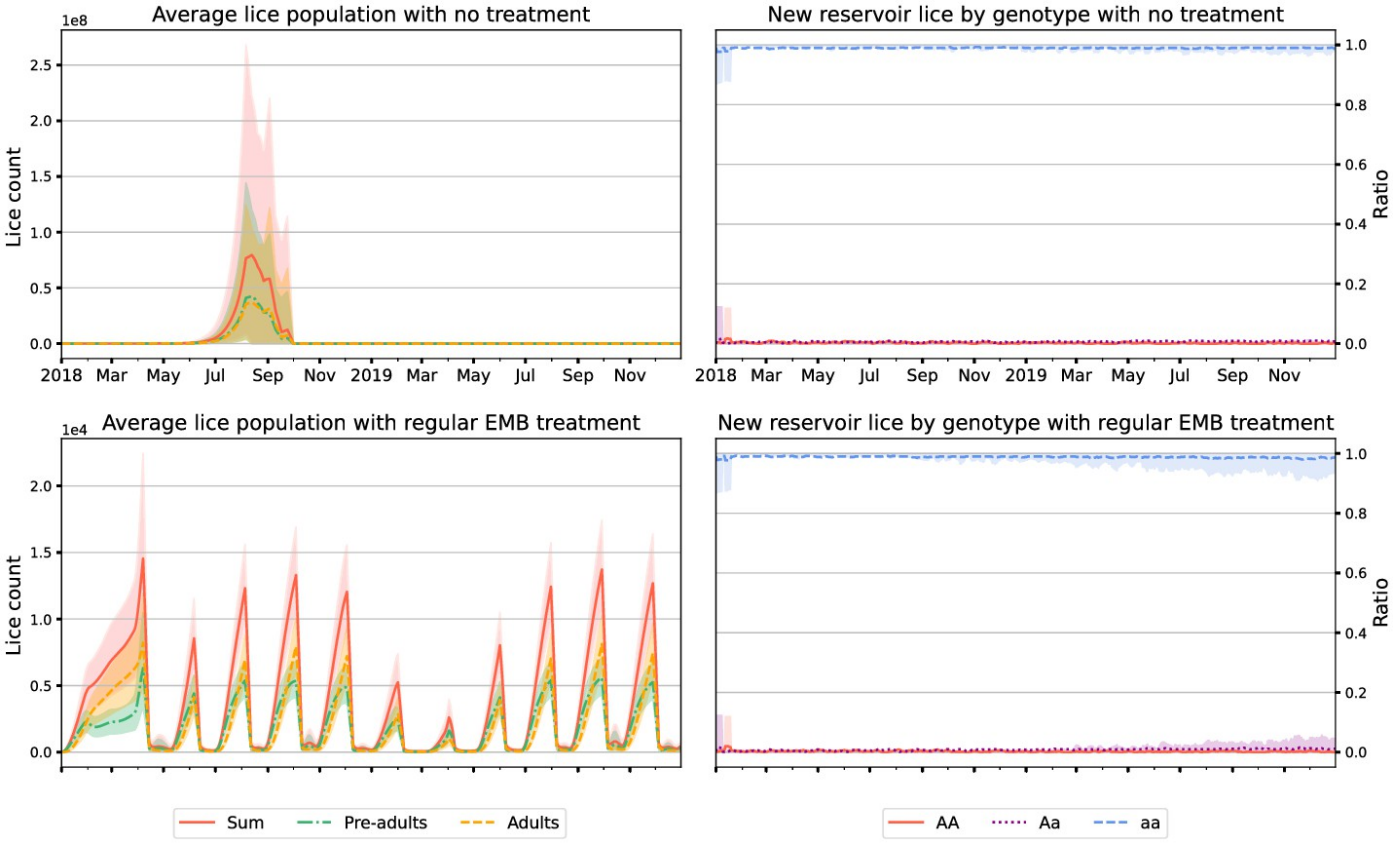
Plots showing the outcomes of 1000 simulations on Loch Fyne with no treatment (top) and scheduled EMB treatment every 30 days starting after the first 5 months (bottom)—in the no-treatment simulations forced fallowing occurred within the first year in all cases. Lice counts are on the left and simulated genotypes in the lice reservoir on the right. AA, Aa, aa represent homogeneous dominant, heterogeneous, and homogeneous recessive genotypes for resistance. Results are from 1000 simulation runs, and the envelopes include 90% of model runs. Note the different axes on the lice number plots.

As we might expect, in scenarios where we do not treat for lice at all we see very high lice numbers (resulting in mandatory fallowing), but no evolution of resistance, Fig 1. Slightly less intuitively, in scenarios with very frequent regular treatment with EMB (e.g. every 30 days) we also see essentially no evolution of resistance: this is because this regime keeps lice within the farms at such low levels that there is little opportunity for evolution (Fig 1).

Both of these scenarios, while advantageous for preventing resistance are ultimately unacceptable for other reasons. In the case of no-treatment the proliferation of lice is damaging—though it could be mitigated by other treatments as they improve. In the case of very frequent scheduled treatment the excessive use of chemical would be unacceptable for environmental reasons.

### 3.2 Defection probability in the Bernoullian setting

**Fig 2:**
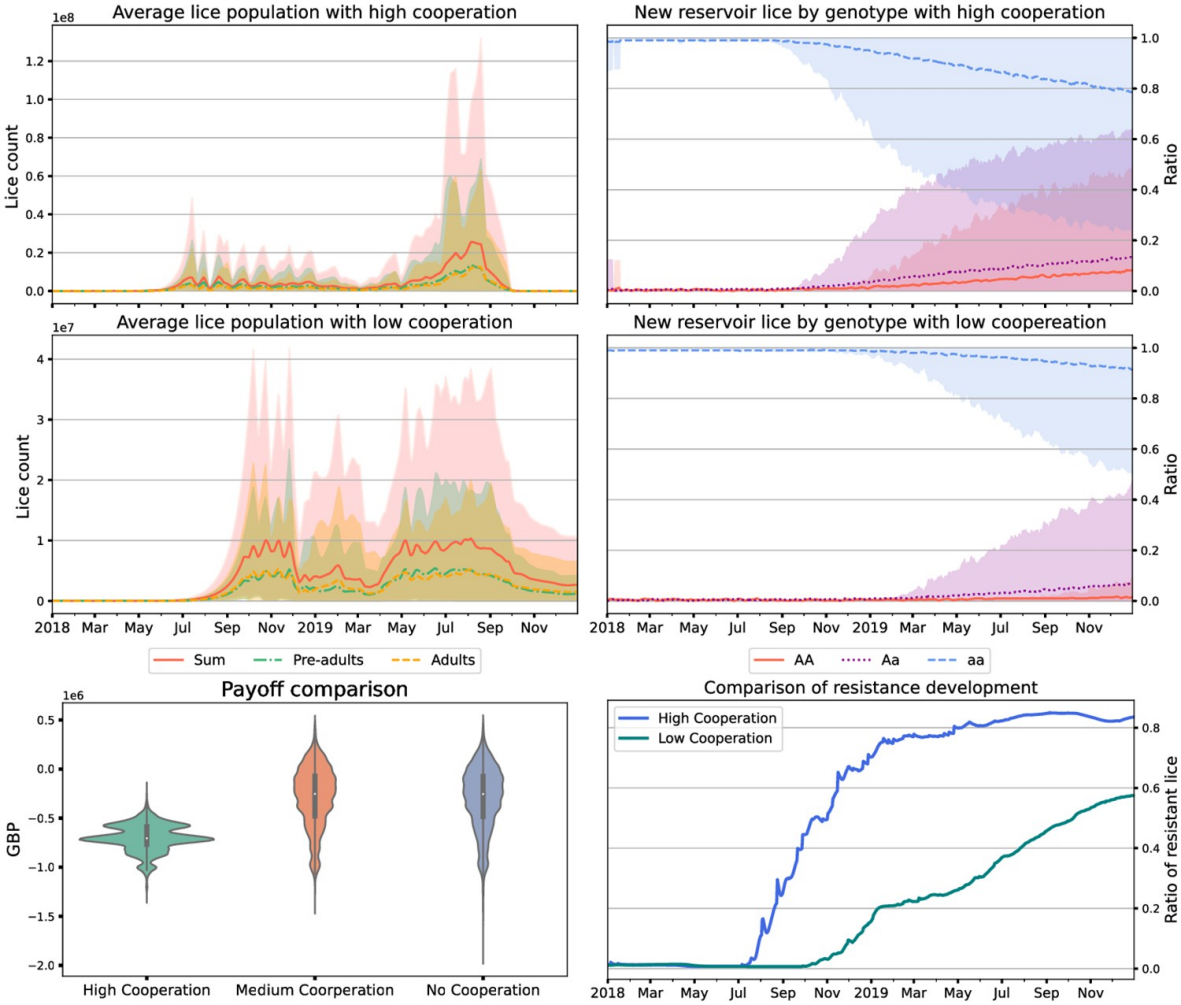
Plots showing the outcomes of 1000 simulations with Bernoullian defection regimes with a defection probability of 0.2 (top row, high cooperation) and 0.8 (middle, low cooperation). Lice counts are shown in the top and middle left, and simulated genotypes in the lice reservoir on the top and middle right. We show violin plots of payoffs on the bottom left over three probabilities of defection (0.2 (left), 0.8 (mid) and 1.0 (right)), and time series of proportion of resistance in lice within the cages on the bottom right. Results are from 1000 simulation runs, and the envelopes include 90% of model runs.

We find that the value of *p* has some impact on the evolution of resistance in the overall system within our modelling setting, Fig 2, with higher levels of cooperation resulting in more resistance. This can be explained by the fact that when farms are more cooperative they treat more frequently, thus driving resistance more quickly if the numbers of sea lice are not sufficiently reduced as surviving lice are more likely to have developed a resistance to treatment. This is in contrast to the previous section where regular treatment did keep lice numbers low enough that there is no opportunity for evolution.

We find that the defection probability has some impact on the payoff derived over the course of the simulation: here intermediate and high defection probabilities give similar results with a low defection probability giving a worse pay-off due to the cost of repeated treatment (Fig 2, bottom left). Note that the payoff function does not incorporate resistance but instead only fish weight/condition and costs of treatment.

### 3.3 Mosaic treatment

**Fig 3:**
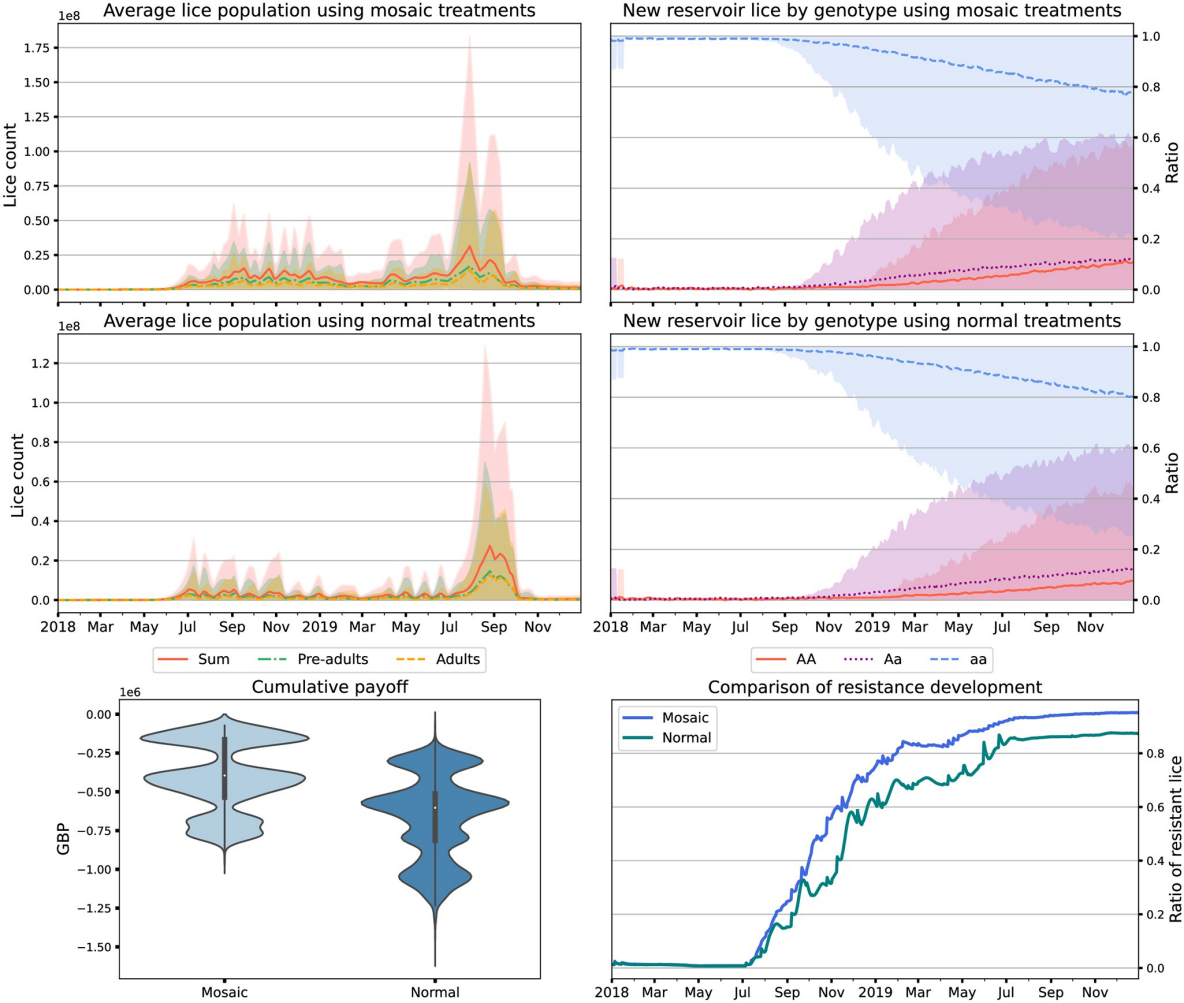
Plots showing the outcomes of 1000 simulations with mosaic treatments (top row) and Bernoullian (’Normal’) treatment regime with no defection (middle row). Lice counts are shown in the top and middle left, and simulated genotypes in the lice reservoir on the top and middle right. We show violin plots of payoffs (in GBP, pounds sterling) on the bottom left, and time series of proportion of resistance in lice within the cages on the bottom right. Results are from 1000 simulation runs, and the envelopes include 90% of model runs.

We find that simulated mosaic treatment results in slightly faster evolution of resistance to EMB than in an all Bernoullian settings (high, *p* = 0.2, medium, *p* = 0.5, and low *p* = 1.0 cooperation) (Fig 3), and slightly higher lice numbers. fIn the mosaic treatment policy, where we cycle through different treatments, there are several periods where we are not treating with EMB but rather with a less effective treatments. Because treatment preferentially removes the least resistant lice (i.e. those having homogeneous recessive genotypes) the periods of less effective treatments creates a selection pressure in the sea-lice that drives up resistance levels. A higher-efficacy alternate treatment (i.e. more effective cleaner fish, a less-stressful physical intervention) may mitigate this result.

### 3.4 Network topology and network position

**Fig 4:**
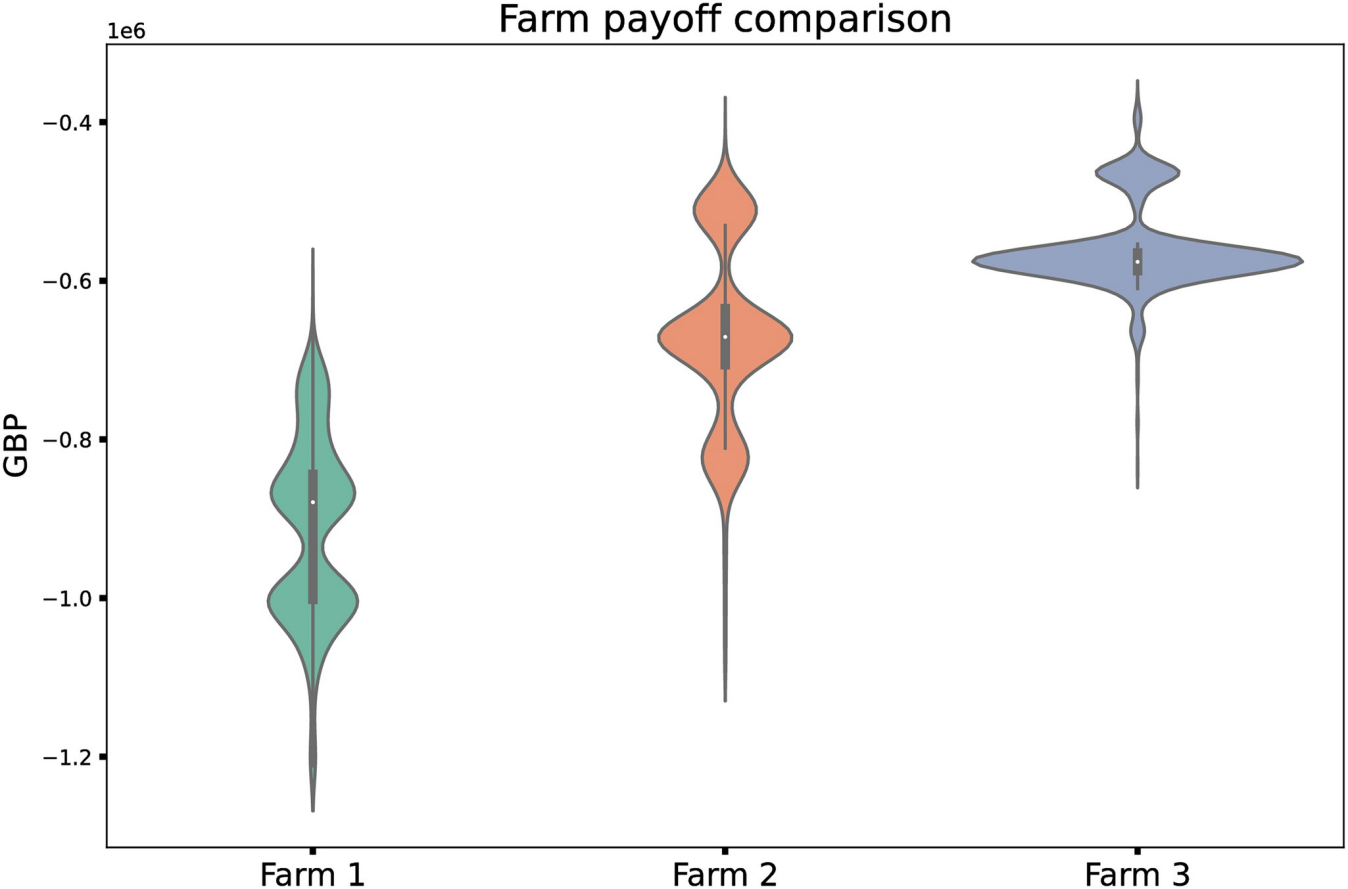
Violin plots showing distributions of final payoff reservoir on the top and middle right. We show violin plots of payoffs (in GBP, pounds sterling) for individual farms within a single loch system in a Bernoullian treatment regime with a defection probability of 0.8. Farms are ordered from left to right by increasing strength of inward connection in the simulated hydrological network: thus Farm 1 expects the fewest incoming lice from other farms, and Farm 3 the most. Distributions are the result of 1000 simulation runs.

Thus far, we have reported mainly aggregate results over an entire simulated sea loch system, but we wish to highlight that in our real-world-inspired networks, we do see differences in lice numbers and payoff between farms in different positions in the network. In particular, farms that have stronger inward connections from more other farms and thus are likely to be recipients of more lice tend to see worse outcomes, and thus overall lower payoffs. In Fig 4 we see this effect, where less-connected farms generally see a better payoffs than more-connected farms.

**Fig 5:**
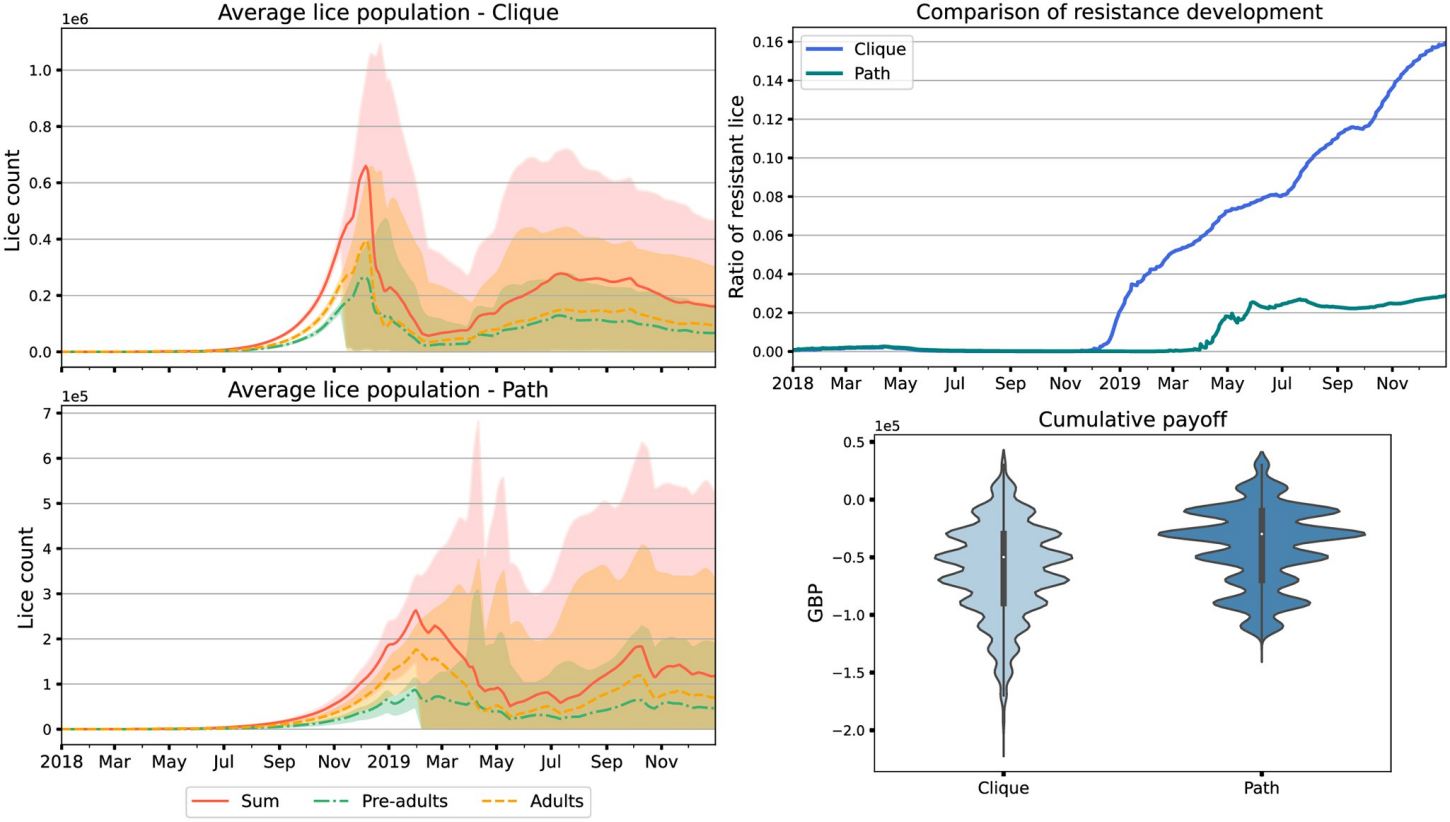
Plots showing the outcomes of 1000 simulations with a Bernoullian regime with a defection probability of 0.2 in different hydrological networks, a clique (top left) and a path (bottom left). We show the time series of the proportion of resistance in lice within cages on the top right and violin plots of payoffs on the bottom right. Results are from 1000 simulation runs, and the envelopes include 90% of model runs.

In addition to our real-world-inspired hydrological networks, we also simulated two theoretical topologies (a completely-connected clique of farms, and a sparse path of farms) to highlight the impact of connection between farms on outcomes in this model. In both cases probability of lice movement is the same for every link in the network. As we might expect, we see more lice and more resistance in the highly-connected case than in the sparsely-connected case, Fig 5. While these topologies may be unrealistic—in particular the fully-connected clique—they highlight an important point that the magnitude and nature of connections between farms can play a role, and thus considering the hydrological connections in a particular setting may be important when planning farm siting and lice control.

### 3.5 Highlights of limitations and suggested future work

We find that the value of the defection probability in the Bernoullian setting has some impact on the evolution of resistance in the overall system within our modelling setting, Fig 2, with higher levels of cooperation resulting in more resistance. This can be explained by the fact that when farms are more cooperative they treat more frequently, thus driving resistance more quickly.

Like all models of complex systems our model has limitations, and to contextualise the interpretation of our results we wish to highlight three of our more important limitations.

The first is uncertainty in the lice life-cycle and treatment model. Our parameters are derived either from published work in similar settings, or from a simple fitting procedure from public Scottish data on lice and fish numbers, but there is nevertheless some uncertainty. In particular, the effectiveness of various treatments is important within our model: if, for example, cleaner fish are much more effective than we have modelled this would impact our regime outcomes. We have designed our model so that if updated estimates of parameters or new treatments become available, it is relatively straightforward to adjust these inputs.

The second is in our choice of genetic mechanism for the evolution of resistance. We have chosen a simple Mendelian scheme in which resistance is dominant, but of course other mechanisms are possible including a many-loci model or a model of maternal inheritance. An investigation of the impact of these different models would be an interesting area of future work.

The third is the relatively simple set of treatment cooperation regimes we have modelled in this first full version of our software. We propose as a future area of work the implementation of more complex systems; e.g. tit-for-tat systems where farms respond to the behaviour of their immediate neighbours, or systems where farmers are optimising under incorrect beliefs about efficacy.

Finally we note that our modelling focused on treatment strategies post-infestation while it has been shown in other models that preventing infestation before lice attach to fish is a more effective strategy than treating already infested salmon [46].

## 4 Conclusion

Using a simulation model of sea lice and salmon farms in a sea loch we have investigated the impacts of farm treatment coordination and hydrological network topology on lice numbers, farm payoff, and the evolution of resistance to treatment in lice. We found that, counter-intuitively, high levels of coordination in lice-threshold-based system may result in faster evolution of resistance due to the larger overall number of treatment applications. This suggests that coordination agreements may need to go beyond a one-treats-all-treat model of cooperation to be most effective.

We also find that more strongly linked networks of farms may result in worse lice outcomes, and more highly-linked farms within a heterogenous network that can therefore receive more lice can have worse outcomes than more isolated farms. It may therefore be important to consider the hydrological connections within a setting when planning lice treatment coordination.

We suggest future work in considering other coordination models and genetic mechanisms for resistance, and highlight the importance of updating this model if updated parameters become available. We also suggest that wild refugia be included in future updates to the model as it may alter the effect of treatment strategies on resistance evolution [34]. It is our sincere hope that our open-source model will be of future use not only to us but to other researchers investigating resistance evolving due to treatment over a network.

## Supporting information

S1 File Details of the model used in this paper. The Supporting Information contains tables of the parameters used in our model with their values and details where this data was obtained. Descriptions of the equations defining the louse lifecycle, egg generation, and egg genotype distributions are also described in detail.
(ZIP)
