# Supplementary material for: A modeling study of the impact of treatment policies on the evolution of resistance in sea lice on salmon farms
Source: PLoS One. 2023 Nov 29;18(11):e0294708. doi: 10.1371/journal.pone.0294708 (PMC10686416; doi:10.1371/journal.pone.0294708)
Supplement: S1 File — The Supporting Information contains tables of the parameters used in our model with their values and details where this data was obtained. Descriptions of the equations defining the louse lifecycle, egg generation, and egg genotype distributions are also described in detail. (ZIP) [file pone.0294708.s001.zip › supporting_info.pdf]

1 Supporting Information

1.1 Data

A total of 9 farms in Loch Fyne (Tarbert South, Rubha Stillaig, Glenan Bay, Meall Mhor, Gob a Bharra, Strondoir Bay, Ardgaddan, Ardcastle, Quarry Point), and 10 in Loch Linnhe (Gorsten, Linnhe, Loch Leven, Kingairloch, Shuna, Lismore North, Loch Creran (B), Walters (East Lismore), Dunstaffnage, Kerrera B) were simulated. The locations of these farms were recorded as eastings and northings coordinates. The 9 farms on Loch Fyne contained 14,10,14,12,10,10,14,9,9 cages respectively [1] initially containing 400,000 fish.

The 10 farms on Loch Linnhe contained 20,24,12,8,10,14,14,9,9,10 respectively [1] initially also containing around 400,000 fish.

Monthly sea temperatures for every cage in each farm were calculated by interpolating the northing of the farm between the mean temperatures at Ardrishaig (northing 685715) and Tarbert (northing 665300). The data for these two locations were taken from `www.seatemperature.org`.

Official reports

Official sea lice count and treatment reports are provided by Marine Scotland, a Scottish Government Agency, and Salmon Scotland Ltd, a body representing Scottish salmon farms. Due to changes in reporting rules in 2015 and 2021, we limited our analysis to the data available between 2016 and 2020. Within this timeframe, we had access to monthly updated mean adult female lice counts per fish across site cages, fish mortality counts and control measures (treatments, biothinning, or farming). Weekly data providing lice counts prior to 2020 are available but largely incomplete or subject to under-reporting, therefore they were not used. These data were used for fitting purposes. Due to lack of granularity, there are limitations in fitting compared to the Norwegian case (section 2.11 in [2]), with respect to no cage count and availability of other substages in the count.

1.2 Louse life Cycle

We calculate the probability of a nauplius(R), chalimus(CH), and pre-adult (PA) louse developing into the next stage after spending n days in a stage using a simplified version of formulae from section 2.2.4 in [2] which assumes a Weibull age distribution within each life-stage. The probability of evolving to the next stage, m, is given as

$$p = \log(2)\delta_s L_n^{\delta_s - 1}\left(\delta m_{10}\left(10/T\right)^{\delta_p}\right)^{-\delta_s} \tag{1}$$

where T is the mean sea-temperature, L_n is the maximum length of time a louse is expected to stay in a particular stage n, the values and descriptions of $\delta p, \delta s, \delta m_{10}$ are parameters for the Weibull distribution and are given in Table 2. We assume that all lice will have an age of zero days in the new evolved stage.

The rate lice evolve to the next stage at an age a is therefore:

$$d_{tfa}^{m} = \min(\log(2)\delta_s a^{\delta_s - 1}\delta_m^{-\delta_s}, 1) \tag{2}$$

Pre-adults (PA) evolve to either adult females or males with equal probability at a rate given in Table 2.

The evolution of lice from the CO to CH stage is only possibly if the louse has managed to attach to a salmon, those that do not attach, die. This approach mimics [2]

Table 1. Overall notation used in this paper.

Symbol	Meaning
f	farm
c	cage
$t, \mathbb{T}$	time and set of timesteps
T	mean sea temperature
N^n, N^{SAL}, N^{CLF}	total gross number of lice at stage n, salmon and cleaner fish
G	allele frequency
W	average salmon weight
Y	expected lice or fish count
e^{EXT}	modifying factor for external recruitment
λ^n	parameters related to lice mortality (stage dependent)
δ^n	lice development rates (stage dependent)
μ^n	lice mortality rates (stage dependent)
γ^n	fish mortality rates (stage dependent)
d	parameters related to lice development (see Table 2)
ζ	policy
$\mathcal{A}_g$	heterozygous allele set of a gene g
$\phi_g^\tau(a)$	phenotype resistance of an allele a of gene g for treatment τ

Table 2. Sea lice development rates per life stage. A louse can only progress from the CO to CH stage if they are attached to a fish. The lifespan of adult females is 80 days. The 10°C median development time for egg stage is taken from [3]. The majority of parameters do not relate to the adult stage, the mortality rates for L5 males is 0.06 and for females is 0.02 [3].

Parameter	Reference	Egg	R	CO	CH	PA
δp, Weibull power constant	[2, 3]	0.338	0.401		1.305	0.866
δs, Weibull shape constant			18.869		7.945	1.643
δm_{10} 10°C median development time	[2, 3]	41.98	8.814		18.934	10.742
Lice dev rates from the ith stage to the next	[3, 4]		0.8	0.47		0.10
L_m, maximum number of days a louse will live in stage m without evolving to the next (days)	[5]		15	10	25	11
Lice natural mortality rates	[2–4]		0.17	0.101	0.008	0.05

and the rate of progression to the next stage is given by

$$d_{tfc}^{CO} = \delta_{0fc}^{CO} + \log N_{tfc}^{SAL} + \delta_1^{CO}(\log(W_{tfc}) - 0.55) \tag{3}$$

where N_{tfc}^{SAL} and W_{tfc} are the number of salmon and average weight (in kg) respectively in cage, c, on farm, f, at time, t. The parameters δ_{0fc}^{CO} and δ_1^{CO} denote the magnitude of the infestation and the variance across farms. We calculated the constants in this expression by fitting the expected loss of CO lice from a farm to reports from Salmon Scotland and Marine Scotland using the Bayesian Optimiser in Ray [6].

The fish mass is determined by fitting a simple logistic curve to FAO data [7] to model the growth of salmon in cages (in kg) as

$$W_{tfc} = \frac{10}{1 + \exp(-0.01 * (t - 475))} \tag{4}$$

In addition to modelling the development of sea lice and their attachment to caged salmon, we explicitly model the genotype of each generation of louse and a mapping to the phenotype for resistance to various treatments. Allowing for a single genotype to

confer resistance [8], we attribute an allele to each egg; dominant (AA), recessive (aa), and heterozygous dominant (Aa). The frequency of a single allele x in the population is represented by $G^m_{tfc}(X = x)$, with the following constraints:

- All the frequencies of the alleles belonging to the same gene must sum to the same gross count.
$$S_g(G^m_{tfc}) = \sum_{x \in \mathbb{A}_{\mathfrak{g}}} G^m_{tfc}(X = x) = N^m_{tfc}$$
- All the frequencies of distinct genes must coincide - i.e. they provide different yet consistent views on the same distribution
$$\forall g \in \mathbb{G}. S_g(G^m_{tfc}) = N^m_{tfc}$$
- All the frequencies must be positive integers.
$$\forall x \in A. G^m_{tfc} \in \mathbb{N}$$

that is, the distribution sums to the actual number of lice and each bin must be discretised. As a shorthand, such sum will also be denoted by S.

During mating, alleles are recombined according to a Mendelian approach with offspring inheriting genotypes from each parent. The phenotype that governs resistance, $\phi^\tau(g)$, to a particular treatment, τ, is modelled as a real number in the range $[0, 1]$ for each gentopye so that we can we can assign partial resistance to heterozygous alleles. This mapping depends on both the type of applied treatment and lice stage. For example, many chemical treatments are effective from chalimus onwards whereas thermolicers are less effective on chalimus themselves [9].

The mortality rate, μ^τ_{tfcg}, the fraction of the population that are killed by a treatment, τ, on farm, f, in cage, c, having genotype, g is calculated as:

$$\mu^\tau_{tfcg} = \begin{cases} 1 - \phi^\tau(g) & \text{if t } \in [t_{fcb} + \delta^\tau, t_{fcb} + \Delta^{dur}] \\ 0 & \text{otherwise} \end{cases}$$

where t represent the current time, t_{fcb} is the time when a treatment was started, δ^{EMB} is the delay of the treatment, Δ^{dur} is the efficacy duration, computed as δ^{dur}/T_{t^0} where T_{t^0} is the average water temperature when the treatment is applied and δ^{dur} is a constant. Thus within the efficacy time frame of a treatment, the mortality rate is computed as 1.0 minus the resistance rate, ϕ^T. We use a Poisson distribution to generate the mortality events and a hypergeometric distribution to choose from which stages to remove lice using the mortality rates for each treatment and phenotype.

1.3 Mating & Reproduction

Mating

Adult female N_{AF} and male N_{AM} lice are randomly assigned to fish according to a negative multinomial distribution. The mean and variance of the number of adult lice on a salmon, assuming we have k lice in stage CH and above (i.e. those that are attached to fish) and n fish in a cage is

$$\begin{aligned} \mu &= n \left[1 - \left(\frac{n-1}{n} \right)^k \right] \\ \nu &= k^2 \frac{n-1}{n^2} \end{aligned} \tag{5}$$

As in [10], we calculate the probability of a mating occurring between two lice on the same host as

$$p_{mating} = 1 - \left(1 + \frac{N_{AM}}{\left(N_{AM} + N_{AF'}\right)\gamma}\right)^{-(1+\gamma)}$$

where $\gamma = \frac{\mu}{\frac{\nu}{\mu} - 1}$, $N_{AF'}$ is the number of adult females that are free to mate, and μ, ν are the mean and variance of lice attached to a salmon (Eq. 5). The (mean) number of matings is $p_{mating}N_{AF'}$. Once an adult female has mated she is no longer free to mate for a period of up to 3 days (the refactory period).

It should be noted that for efficiency reasons SLIM does not keep track of when lice enter and leave the refractory stage. Reducing this to a classical M/M/1 queue problem, we use Little's law to approximate the average queue load, assuming we know the arrival rate λ^r and the waiting time (3 days). Since the average load rate can fluctuate dramatically across different stages it is estimated with a simple AR(1) model over the last 3 days:

$$\begin{aligned}\lambda^r_{0fc} &= 0\\ \lambda^r_{tfc} &= \frac{2}{3}\lambda^r_{(t-1)fc} + \frac{1}{3}n^{AF}_{t-1}\\ N^{AF'} &= \mathrm{round}(N^{AF}\lambda^r_{tfc})\end{aligned}$$

Egg generation

The number of eggs, N^{egg}_{tfc}, produced after each mating is defined in a similar way to [2] (section 2.2.6) and follows a power law parameterised on the (virtual) age distribution, A.

$$N^{Egg}_{tfc} = \beta^r_0 \left(a+1\right)^{\beta^r_1} A$$

We set the values of β^r_0 and β^r_1 to 172.5 and 0.2 respectively, as in [2], and A is the age distribution of females that have mated. The distribution is calculated from equations 4,6,8 in [2]. The expected date eggs hatch is calculated using the regression formula in [3] (a Belehrádek function)

$$\tau_E = \left(\frac{\beta_1}{\left(\langle T\rangle - 10 + \beta_1 * \beta_2\right)}\right)^2$$

where $\langle T\rangle$ is the average temperature centered at 10 degrees, β_1, β_2 are the parameters of the Belehrádek function. To allow for randomness in the hatching time we draw the expected hatching date from a Poisson distribution with a mean of τ_E and maintain a list of expected hatching dates for each cage. At each time-step, we inspect this list and add new R lice accordingly.

Eggs can either be transmitted between farms or lost. Additionally, the external pressure will absorb part of these lost eggs and introduce new hatched lice into the system every day.

The majority of lice hatching from eggs are typically lost, some are transported to neighbouring farms via sea currents and very few are reintegrated into the reservoir. Knowing the locations of each farm we calculate the Euclidean distance between a pair of farms, d_{ij}, and is the probability that an egg leaving farm, i, reaches farm j, r_{ij} from [11]. The time, t', and number of eggs reaching farm j, N^{Egg}_j, if N^{Egg}_i eggs leave

farm i is

$$t' = t + \mathrm{Poisson}(d_{i,j})$$
$$N^{Egg} = \min(\mathrm{Poisson}(N_i^{Egg} \times r_{i,j}),\ N_i^{Egg})$$

Egg genotype distribution

Assume n^{AM} and n^{AF} lice have been selected in the mating process each with a distribution of alleles for each gene.

Let aa, AA, Aa be the different heterozygotic alleles arising for a particular gene.

By using the Mendelian rules on mating, and noting that the total number of possible matings is $c^E = n^{AF} n^{AM}$, we consider the following three cases:

The number of homozygously recessive pairs arise from mating between two recessive partners, or from a recessive and a heterozygous partner. In the latter case only a half of these will yield the recessive gene. Thus:

$$c_{aa}^{Egg} = \left(g_{aa}^{AF} + \frac{1}{2} g_{Aa}^{AF}\right)\left(g_{aa}^{AM} + \frac{1}{2} g_{Aa}^{AM}\right)$$

The number of homozygously dominant pairings are similarly defined:

$$c_{AA}^{Egg} = \left(g_{AA}^{AF} + \frac{1}{2} g_{Aa}^{AF}\right)\left(g_{AA}^{AM} + \frac{1}{2} g_{Aa}^{AM}\right)$$

The heterozygous pairings can be deduced by subtracting the total number of homozygous pairs from the total:

$$c_{Aa}^{Egg} = c^E - \left(c_{aa}^{Egg} + c_{AA}^{Egg}\right)$$

From these we create a multinomial probability distribution for the alleles with genotype c_g^{Egg} from which we select the allele for the produced eggs

$$p_g^{Egg} = c_g^{Egg} / c^{Egg}$$
$$G^{Egg} \sim \mathrm{Multinomial}(N^{Egg} | p^{Egg})$$

Farm-to-farm movements

Using the Scottish Shelf model [11], we calculated matrices for the average time taken for eggs to travel between two sites, $T_{i,j}$, and the probabilities of eggs arriving at the destination, S_i. Since eggs are lost during travel the survival matrix is not stochastic. Eggs may hatch during travel so we consider arriving lice to be in the R or CO stage with equal probability depending on the travel time.

The number of eggs/lice arriving at a farm i within a time T_{ij} having departed farm j at time t is calculated from

$$N_i^{Egg}(t + T_{ij}) \sim \mathrm{Multinomial}(N_j^{Egg}(t) | p = S_i)$$

External pressure

The external pressure can be thought as a generator of R lice. The number of lice entering a farm is modelled as a linear formula:

$$N^{R^{EXT}}(t) = r_0^{EXT} + r_1^{EXT} \ \langle N^{Egg}(\tau^{EXT}) \rangle$$

where $\langle N^{Egg}(\tau^{EXT})\rangle$ is a rolling average of the number of released offspring in the whole loch between $t-\tau^{EXT}$ and $t-1$, and τ^{EXT} is the number of days over which we consider the rolling average. r_0^{EXT}, r_1^{EXT} are linear model coefficient (intercept and slope) that fit the data.

The allele distribution is modelled as a Bayesian Multinomial distribution

$$G_g(n_t^{REXT}) = \text{Multinomial}(N^{R^{EXT}}(t)|p_{mtg}^{EXT})$$

where p_{mtg}^{EXT} is the probability distribution of the genotypes g of the lice in stage m in the loch at time t. p_{mtg}^{EXT} is a Dirichlet distribution which is updated at each time step.

Natural Mortality of Sea Lice

We consider natural lice death as a constant rate across all stages, m, in a way akin to [3] rather than [2].

$$\mu^m = k$$
$$n^{\mu^m} = \text{Poisson}(N_k^m \mu^m)$$

Natural mortality is considered irrespective of the genotype, meaning each allele is equally likely to be removed.

Louse Mortality due to Treatment

Three different models for treatment mortality are modelled within SLIM: chemical, mechanical and biological.

- **Chemical treatment mortality** is modelled after [2], but with the addition that the resistant rates related to with lice genomics. The mortality due to treatment, e.g. EMB, applied at time t_{fc} to cage c on farm f for a louse with genotype g is

$$\mu_{tfcg}^{EMB} = \begin{cases} 1-\phi^{EMB}(g) & \text{if t } \in [t_{fc}+\delta^{EMB}, t_{fc}+\Delta^{dur}] \\ 0 & \text{otherwise} \end{cases}$$

  where ϕ^{EMB} is the phenotype resistance corresponding to the given genotype g, modelled as a continuous variable within the range $[0, 1]$. Δ^{dur} is length of time the treatment is effective, and δ^{EMB} is the delay between the application of the treatment and its impact on lice.

  Without loss of generality, other chemical treatments behave similarly. The only notable exception is H_2O_2, not used in our simulations, which assumes Δ^{dur} to be temperature-independent.

- **Mechanical treatment mortality** is modelled after a non-linear function fitted to lice mortality reports in [12] for thermolicer and hydrolicer treatments.

- **Cleaner fish treatment** is defined a simple exponential curve as in [2],

$$\mu_{tfc}^{CF} = 1 - e^{-\lambda^{clf} \frac{N_{tfc}^{CLF}}{N_{tfc}^{SAL}}}$$

1.4 Fish Mortality

In principle, lice tend not to cause their host death unless in excessive loads or for long periods of time (which may facilitate the spread of pancreatic infections or Gill's Disease). Due to regulation on allowed lice aggregation [13], treatment and culling are more likely causes of death, but nevertheless we consider this important.

Natural Fish Death

Background fish mortality is assumed to be a simple constant $\mu_b^{SAL} =$ constant, with the constant being taken from [14].

Mortality due to lice

Fish mortality due to lice depends uniquely on pathogenic lice load, which comprises PA and A stages. We use a simple sigmoid-based approach as done (and with coefficients taken from) [15]:

$$\begin{aligned} x_{tfc}^{path} &= \frac{N^{PA} + N^{A}}{N^{SAL}} \\ \chi_{tfc}^{path} &= e^{w_{pfm} - w_{pfk} x_{tfc}^{path}} \\ \mu_{path}^{SAL} &= 1 - \frac{\chi_{tfc}^{path}}{1 + \chi_{tfc}^{path}} \end{aligned}$$

Mortality due to treatment

Despite being the largest cause of fish death, to our knowledge no clear model for fish mortality due to treatment has been developed so far. We fit a simple quadratic curve to model mortality using the data in [9].

Lice Mortality due to fish death

Assuming k fish died, we estimate how many lice were on them by multiplying the expected load found in Eq. 1.3.

$$\begin{aligned} n^{inf} &= N^{CH} + N^{PH} + N^{AM} + N^{AF} \\ n^{infSAL} &= \mathbb{E}[load(n^{inf})|N^{SAL}] \\ n_m^{aff} &= k n_m^{inf} / n^{infSAL} \quad m \in \{CH, PH, AM, AF\} \\ m^{aff} &= \text{largest\_remainder}(n^{aff}) \end{aligned} \tag{6}$$

Of these, male pre-adults (assuming 1/2 of all PA) and AM are eligible to survival, other lice will die.

$$\begin{aligned} \mu_h^{CH} = m_{CH}^{aff} \qquad \mu_h^{AF} &= m_{AF}^{aff} \\ \mu_h^{PA} &= \frac{1}{2}\mu_h m_{PA}^{aff} \\ \mu_h^{AM} &= \mu_h m_{AM}^{aff} \end{aligned} \tag{7}$$

With μ_h being our detachment rate.

Once the mortality rates μ are computed for each genotype, we use a Poisson distribution to generate the mortality events and a hypergeometric distribution to choose from which stages to remove lice.

Since EMB affects lice attachment only the stages from CH onward are affected. Cleaner fish is considered effective only from PA stages onwards. Physical treatments affect stages CH onwards.

References

1. Marine Scotland. Marine Scotland Data Portal;. Available from: `https://marine.gov.scot/data/marine-scotland-data-portal`.
2. Aldrin M, Huseby R, Stien A, Grøntvedt R, Viljugrein H, Jansen P. A stage-structured Bayesian hierarchical model for salmon lice populations at individual salmon farms - Estimated from multiple farm data sets. Ecological Modelling. 2017;359. doi:10.1016/j.ecolmodel.2017.05.019.
3. Stien A, Bjørn P, Heuch P, Elston D. Population dynamics of salmon lice Lepeophtheirus salmonis on Atlantic salmon and sea trout. Marine Ecology Progress Series. 2005;290:263–275. doi:10.3354/meps290263.
4. Tucker CS, Sommerville C, Wootten R. Does size really matter? Effects of fish surface area on the settlement and initial survival of Lepeophtheirus salmonis, an ectoparasite of Atlantic salmon Salmo salar. Diseases of Aquatic Organisms. 2002;49(2):145–152. doi:10.3354/dao049145.
5. Heuch P, Mo T. A model of salmon louse production in Norway: effects of increasing salmon production and public management measures. Diseases of Aquatic Organisms. 2001;45:145–152. doi:10.3354/dao045145.
6. Moritz P, Nishihara R, Wang S, Tumanov A, Liaw R, Liang E, et al.. Ray: A Distributed Framework for Emerging AI Applications; 2017. Available from: `https://arxiv.org/abs/1712.05889`
7. Food, of the United Nations AO. Aquaculture Feed and Fertilizer Resources Information System; 2022. Available from: `https://www.fao.org/fishery/affris/species-profiles/atlantic-salmon/growth/en/`.
8. Jensen EM, Sevatdal S, Bakke MJ, Kaur K, Horsberg TE. A selection study on a laboratory-designed population of salmon lice (Lepeophtheirus salmonis) using organophosphate and pyrethroid pesticides. PLoS ONE. 2017);12(5):e0178068. doi:10.1371/journal.pone.0178068.
9. Overton K, Dempster T, Oppedal F, Kristiansen TS, Gismervik K, Stien LH. Salmon lice treatments and salmon mortality in Norwegian aquaculture: a review. Reviews in Aquaculture. 2019;11(4):1398–1417. doi:10.1111/raq.12299.
10. Cox R, Groner ML, Todd CD, Gettinby G, Patanasatienkul T, W RC. Mate limitation in sea lice infesting wild salmon hosts: the influence of parasite sex ratio and aggregation. Scientific Reports. 2017;8(12):e02040. doi:10.1002/ecs2.2040.
11. Mccollin T, Murray RO, McGregor H. The Scottish Shelf Model. Part 5: Wider Loch Linnhe Sub-Domain: Scottish Marine and Freshwater Science Vol 7 No 7; 2016.
12. Roth B. Deworming of salmon with Optilice: Effect on deworming and fish welfare. https://nofima.com/publication/1408716/: Nofima AS; 2016. 59/2016.
13. Aquaculture and Fisheries (Scotland) Act 2013; 2013. Available from: `https://www.legislation.gov.uk/asp/2013/7/contents`.
14. Munro LA, Wallace IS. Scottish fish farm production survey 2016; 2017. Available from: `https://www.gov.scot/publications/scottish-fish-farm-production-survey-2016/`.

15. Vollset KW. Parasite induced mortality is context dependent in Atlantic salmon: insights from an individual-based model. Scientific Reports. 2019;9(1):17377. doi:10.1038/s41598-019-53871-2.
